# Theoretical basis to measure the impact of short-lasting control of an infectious disease on the epidemic peak

**DOI:** 10.1186/1742-4682-8-2

**Published:** 2011-01-26

**Authors:** Ryosuke Omori, Hiroshi Nishiura

**Affiliations:** 1 Department of Biology, Faculty of Sciences, Kyushu University, 6-10-1 Hakozaki, Higashi-ku, Fukuoka 812-8581, Japan; 2 PRESTO, Japan Science and Technology Agency, 4-1-8 Honcho, Kawaguchi, Saitama 332-0012, Japan; 3 Theoretical Epidemiology, University of Utrecht, Yalelaan 7, Utrecht, 3584 CL, The Netherlands; 4 School of Public Health, The University of Hong Kong, Pokfulam, Hong Kong Special Administrative Region, PR China; 5 Centre for Infectious Diseases, University of Edinburgh, Kings Buildings, West Mains Road, Edinburgh EH9 3JT, UK

## Abstract

**Background:**

While many pandemic preparedness plans have promoted disease control effort to lower and delay an epidemic peak, analytical methods for determining the required control effort and making statistical inferences have yet to be sought. As a first step to address this issue, we present a theoretical basis on which to assess the impact of an early intervention on the epidemic peak, employing a simple epidemic model.

**Methods:**

We focus on estimating the impact of an early control effort (e.g. unsuccessful containment), assuming that the transmission rate abruptly increases when control is discontinued. We provide analytical expressions for magnitude and time of the epidemic peak, employing approximate logistic and logarithmic-form solutions for the latter. Empirical influenza data (H1N1-2009) in Japan are analyzed to estimate the effect of the summer holiday period in lowering and delaying the peak in 2009.

**Results:**

Our model estimates that the epidemic peak of the 2009 pandemic was delayed for 21 days due to summer holiday. Decline in peak appears to be a nonlinear function of control-associated reduction in the reproduction number. Peak delay is shown to critically depend on the fraction of initially immune individuals.

**Conclusions:**

The proposed modeling approaches offer methodological avenues to assess empirical data and to objectively estimate required control effort to lower and delay an epidemic peak. Analytical findings support a critical need to conduct population-wide serological survey as a prior requirement for estimating the time of peak.

## Background

The influenza A (H1N1-2009) pandemic began in early 2009, and rapidly spread worldwide. Mathematical epidemiologists characterized the epidemic and provided key insights into its dynamics from the earliest stages of the pandemic [1]. The transmission potential was quantified shortly after the declaration of emergence [2-6], while statistical estimation and relevant discussion of epidemiological determinants were underway before substantial numbers of cases were reported in many countries [1].

Prior to the pandemic, many countries issued the original pandemic preparedness plans and guidelines, aiming to instruct the public and to advocate community mitigation. The goals of the mitigation have been threefold; (a) to delay epidemic peak, (b) to reduce peak burden on hospitals and infrastructure (by lowering the height of peak) and (c) to diminish overall morbidity impacts [7]. To assess these aspects under different intervention scenarios, various modeling studies have been conducted (e.g. [8-10]), most notably, by simulating the detailed influenza transmission dynamics.

Although simulations have aided our understanding of expected dynamics in realistic situations and in different scenarios, analytical methods that objectively determine the required control effort and that make statistical inference (e.g. evaluation of empirically observed delay) have yet to be developed. Focus on epidemic peak (relating to mitigation goals (a) and (b) above) has been particularly understudied. Goal (c), on the other hand, is readily formulated in terms of the so-called final epidemic size. The time delay of a major epidemic (such as that resulting from international border control) has been explored using simplistic modeling approaches [11,12]; however, the height and time of an epidemic peak involve nonlinear dynamics, rendering analytical approaches difficult. Despite the mathematical complexity, goals (a) and (b) can be more readily understood from empirical data during early epidemic phase than can goal (c), because an explicit understanding of goal (c) in the presence of interventions requires knowledge of the full epidemiological dynamics over the entire epidemic period.

In the present study, we present a theoretical basis from which the impact of an early intervention on the height and time of epidemic peak may be assessed. As a special case, we consider a scenario in which intervention is implemented only briefly during the early epidemic phase (e.g. unsuccessful containment). We employ a parsimonious epidemic model with homogeneously mixing assumption, because nonlinear epidemic dynamics involve a number of analytical complexities. As a first step towards understanding epidemiological factors that influence the epidemic peak, leading to the eventual statistical inference of relevant effects, we seek fundamental analytical strategies to evaluate the impact of short-lasting control on epidemic peak using the simplest epidemic model [13]. For our model to become fully applicable and to more closely match empirical data, a number of extensions are required. We discuss ways by which these extensions can be practically realized.

## Methods

### Study motivation

We first present our study motivation. During the early epidemic phase of the 2009 pandemic, many countries initially enforced strict countermeasures to locally contain the epidemic. Early intervention includes, but is not limited to, quarantine, isolation, contact tracing and school closure. Nevertheless, once it was realized that a major epidemic was unavoidable, regions and countries across the world were compelled to downgrade control policy from containment to mitigation. Although mitigation also involves various countermeasures (and indeed, mitigation originally intends to achieve the above mentioned goals (a)-(c)), one desires to know the effectiveness of the unsuccessful containment effort. Among its many outcomes, the present study focuses on the height and time of epidemic peak.

The applicability of our theoretical arguments is not restricted to the switch of control policy. In many Northern hemisphere countries, the start of the major epidemic of H1N1-2009 (which may or may not have been preceded by early stochastic phase) corresponds to the summer school holiday period. Adults also take vacation over a part of this period. In addition to strategic school closure as an early countermeasure against influenza [14,15], school holiday is known to suppress the transmission of influenza [16], mainly because transmission tends to be maintained by school-age children [2,17-19]. Following this trend, a decline in instantaneous reproduction number has been empirically observed during the summer holiday period of the 2009 pandemic [20]. Transmission resumes once a new semester starts. The effectiveness of the summer holiday period in lowering and delaying the epidemic peak is, therefore, a matter of great interest.

**Figure 1:**
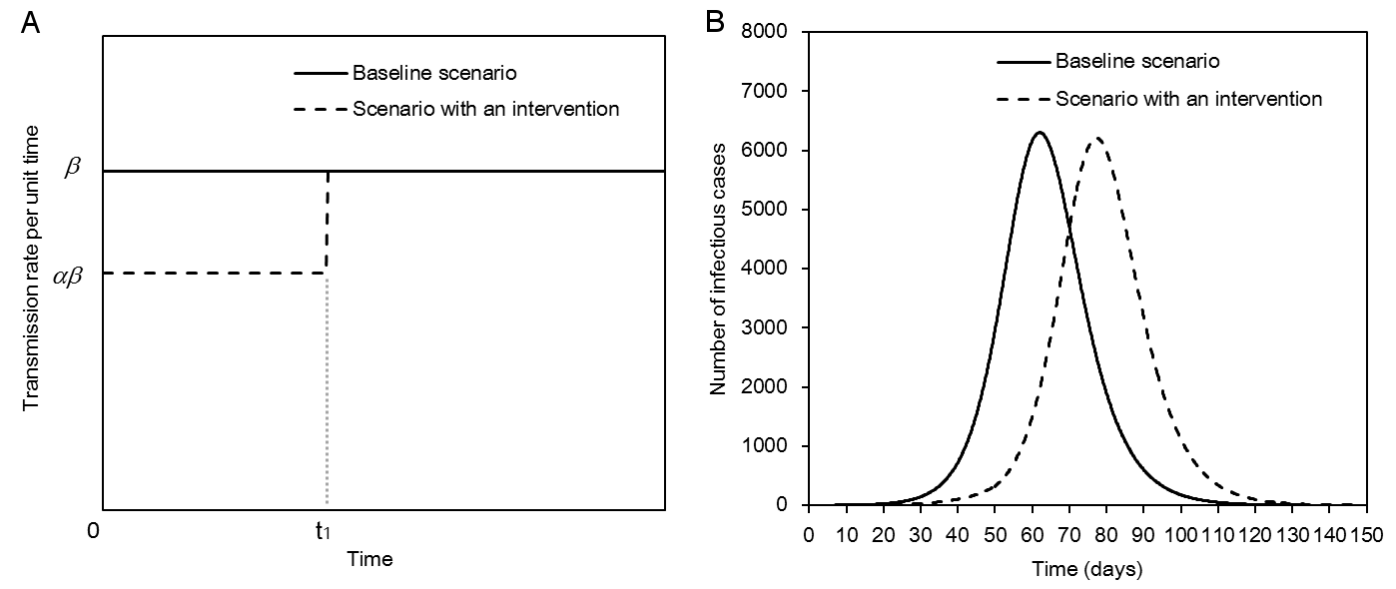
**A scenario for time-dependent increase in the transmission potential**. **A**. Time dependent increase in the transmission rate. In the absence of intervention (baseline scenario), the transmission rate is assumed to be constant *β *over time. In the presence of early intervention, the transmission rate is reduced by a factor *α *(0 ≤ *α *≤ 1) over time interval 0 to *t*_1_. We assume that the product *αβ *ileads to super-critical level (i.e. *αR*(0) *>*1 where *R*(0) is the reproduction number at time 0), and *t*_1 _occurs before the time at which peak prevalence of infectious individuals in the absence of intervention is observed. **B**. A comparison between two representative epidemic curves (the number of infectious individuals) in a hypothetical population of 100,000 individuals. *R*(0) = 1.5, *α *= 0.90 and *t*_1 _= 50 days. The epidemic peak in the presence of short-lasting control is delayed, and the height of epidemic curve is slightly reduced, relative to the case in which control measures are absent.

Both questions are addressed by considering time-dependent increase in the transmission rate. Let *β *be the transmission rate per unit time in the absence of an intervention of interest (or during the mitigation phase in the case of our first question). Due to intervention (or school holiday) in the early epidemic phase, *β *is initially reduced by a factor *α *(0 ≤ *α *≤ 1) until time *t*_1 _(Figure 1A). Though transmission rate abruptly increases at time *t*_1 _when the control policy is eased or when the new school semester starts, we observe a reduced height of, and a time delay in, the epidemic peak compared to the hypothetical situation in which no intervention takes place (Figure 1B). More realistic situations may be envisaged (e.g. a more complex step function or seasonality of transmission), but we restrict ourselves to the simplest scenario in the present study.

### Epidemic model

Here we consider the simplest form of Kermack and McKendrick epidemic model [13], formulated in terms of ordinary differential equations. The following assumptions are made: (i) the population is homogeneously mixing, (ii) the epidemic occurs in a population in which the majority of individuals are susceptible, (iii) the time scale of the epidemic is sufficiently shorter than the average life expectancy at birth of the host, and we ignore the background demographic dynamics, (iv) the epidemic occurs in a closed constant population without immigration and emigration again justified based on time scale, and (v) once an infected individual recovers, he/she becomes completely and permanently immune against further infections. Let the numbers of susceptible, infectious and recovered individuals at calendar time *t *be *S*(*t*), *I*(*t*) and *U*(*t*), respectively. We use the notation *U*(*t*) for recovered individuals to avoid confusion with the instantaneous reproduction number at calendar time *t*, *R*(*t*). The population size *N *remains constant over time (*N *= *S*(*t*) + *I*(*t*) + *U*(*t*)). The so-called SIR (susceptible-infected-recovered) model is written as

(1) $$\begin{array}{l} \frac{dS\left(t\right)}{dt}=-\gamma R\left(t\right)I\left(t\right),\\ \frac{dI\left(t\right)}{dt}=\gamma R\left(t\right)I\left(t\right)-\gamma I\left(t\right),\\ \frac{dU\left(t\right)}{dt}=\gamma I\left(t\right), \end{array}$$

where *R*(*t*) is the instantaneous reproduction number (i.e., the average number of secondary cases generated by a single primary case at calendar time *t*) and γ is the rate of recovery. Given time-dependent transmission rate *β*(*t*) and susceptible population size *S*(*t*) at time *t*, *R*(*t*) is assumed to be given by

(2) $$R\left(t\right)=\frac{\beta \left(t\right)S\left(t\right)}{\gamma }.$$

Although *β*(*t*) will be dealt with as a simple step function in the following analysis, we use the general notation to motivate future analysis of more complex time-dependent dynamics. We assume that an epidemic starts at time 0 with an initial condition (*S*(0), *I*(0), *U*(0)) = (*S*_0_, *I*_0_,*U*_0_) where *I*_0 _= 1 and *U*_0_/*N ≈ *0, i.e. an epidemic occurs in a population in which the majority of individuals are susceptible at *t *= 0. Under this initial condition, we consider two different scenarios for *R*(*t*). First, a hypothetical scenario in which no intervention takes place, i.e.

(3) $$R\left(t\right)=\frac{\beta S\left(t\right)}{\gamma },$$

which is hereafter referred to as the baseline scenario. Second, we consider an observed scenario in which an intervention takes place during the early stage of the epidemic. Let *t*_1 _and *t*_*m*,0 _be calendar times at which the intervention terminates, and at which a peak prevalence of infectious individuals is observed in the absence of intervention, respectively. As mentioned above, we assume that the intervention reduces the reproduction number by a factor *α *(0 ≤ *α *≤ 1) for 0 ≤ *t < t*_1_. For *t *≥ *t*_1_, we assume that the transmission rate is recovered to *β *as in (3).

(4) $$R\left(t\right)=\{\begin{array}{cc} \frac{\alpha \beta S\left(t\right)}{\gamma } & \text{for}\;\text{0}\leq t<t_{1},\\ \frac{\beta S\left(t\right)}{\gamma } & \text{for}\;t\geq t_{1}. \end{array}$$

We assume *t*_1 _<*t*_*m*,0_, i.e. we consider a scenario in which transmission rate recovers before the time at which peak prevalence is observed in baseline scenario. We further assume that *R*(*t*) *>*1 for *t < t*_1_. That is, the efficacy *α *of an intervention effort (or summer holiday) is by itself not sufficient to contain the epidemic.

To illustrate our modeling approaches, we consider the transmission dynamics of pandemic influenza (H1N1-2009), ignoring the detailed epidemiological characteristics (e.g. pre-existing immunity, realistic distribution of generation time and the presence of asymptomatic infection). The initial reproduction number in the absence of interventions *R*(0) is assumed to be 1.4 [2]. Given that expected values of empirically estimated serial interval ranged from 1.9 to 3.6 days [2,5,21-23], the mean generation time 1/*γ *is assumed to be 3 days [24,25].

Our study questions are twofold. First, we aim to quantify the decline in peak prevalence (*I*(*t*)/*N*) due to a short-lasting intervention. The peak prevalence of the intervention scenario is always smaller than that of baseline scenario (see below), and we show that this difference can be analytically expressed. Second, we are interested in the time delay in observing peak prevalence in the presence of intervention. We develop an approximate strategy to quantify the difference in times of peak between baseline and intervention scenarios.

### Difference in peak prevalence

We move on to consider estimates of peak prevalence in two scenarios. For mathematical convenience, we use the prevalence of infectious individuals (*I*(*t*)/*N*) to consider the epidemic peak. The peak prevalence of infectious individuals is preceded by peak incidence (*γR*(*t*)*I*(*t*)/*N*) by approximately the mean infectious period of 1/*γ *days. As was realized elsewhere [26], analysis of prevalence is easier than that of incidence. Beginning with two sub-equations of system (1), we have

(5) $$\frac{dI\left(t\right)}{dS\left(t\right)}=-1+\frac{1}{R\left(t\right)}.$$

Note that *R*(*t*) is a function of *S*(*t*). Integrating (5) in baseline scenario, we obtain [27]

(6) $$I\left(t\right)=I_{0}+S_{0}-S\left(t\right)+\frac{\gamma }{\beta }\ln\frac{S\left(t\right)}{S_{0}}.$$

A theoretical condition for the observation of peak prevalence at time *t*_*m*,0 _is *dI*(*t*_*m*,0_)/*dt *= 0, or equivalently, *R*(*t*_*m*,0_) = 1. As evident from equation (2), this condition satisfies *S*(*t*_*m*,0_) = *γ*/*β*. The peak prevalence *I*(*t*_*m*,0_)/*N *is then given by [28]

(7) $$\begin{array}{c} \frac{I\left(t_{m,0}\right)}{N}\;\;=\;\;\frac{I_{0}+S_{0}}{N}-\frac{\gamma }{\beta N}\left(1+\ln\frac{S_{0}\beta }{\gamma }\right)\\ \approx \;\;1-\frac{S_{0}}{R\left(0\right)N}\left(1+\ln R\left(0\right)\right). \end{array}$$

Note that *S*_0_/*R*(0)*N *represents the proportion yet to be infected and *S*_0 _ln *R*(0)/*R*(0)*N *is the proportion removed at time *t*_*m*,0_. Equation (7) indicates that the peak prevalence of SIR model is determined by the initial condition and the transmission potential *R*(0). It should be noted that *S*_0_/*R*(0) can be replaced by *γ*/*β*, and thus, *I*(*t*_*m*,0_) is independent of initial condition for *U*_0 _= 0 (a special case).

In the intervention scenario, equation (6) with replacement of *β *by *αβ *applies for *t < t*_1_.

(8) $$I\left(t_{1}\right)+S\left(t_{1}\right)=I_{0}+S_{0}+\frac{\gamma }{\alpha \beta }\ln\frac{S\left(t_{1}\right)}{S_{0}},$$

which provides another initial condition at time *t *= *t*_1 _for *t *≥ *t*_1_. That is, we can also employ (6) to compute peak prevalence for *t *≥ *t*_1 _with initial condition (*S*(*t*_1_), *I*(*t*_1_),*U*(*t*_1_)). Again, a condition to observe peak prevalence at time *t*_*m*,1 _is *R*(*t*_*m*,1_) = 1, which gives *S*(*t*_*m*,1_) = *γ*/*β*. The peak prevalence *I*(*t*_*m*,1_)/*N *of the intervention scenario is given by

(9) $$\begin{array}{c} \frac{I\left(t_{m,1}\right)}{N}\;\;=\;\;\frac{I\left(t_{1}\right)+S\left(t_{1}\right)}{N}-\frac{\gamma }{\beta N}\left(1+\ln\frac{S\left(t_{1}\right)\beta }{\gamma }\right)\\ =\;\;\frac{I\left(t_{1}\right)+S\left(t_{1}\right)}{N}-\frac{S_{0}}{R\left(0\right)N}\left(1+\ln\frac{R\left(0\right)S\left(t_{1}\right)}{S_{0}}\right). \end{array}$$

Note that *R*(0) in the above equation refers to *βS*_0_/*γ *(i.e. we use *R*(0) in our baseline scenario to permit an explicit comparison between the two scenarios). Inserting right-hand side of (8) into (9), we obtain

(10) $$\begin{array}{c} \frac{I\left(t_{m,1}\right)}{N}\;\;=\;\;\frac{I_{0}+S_{0}}{N}+\frac{S_{0}}{\alpha R\left(0\right)N}\ln\frac{S\left(t_{1}\right)}{S_{0}}-\frac{S_{0}}{R\left(0\right)N}\left(1+\ln\frac{R\left(0\right)S\left(t_{1}\right)}{S_{0}}\right)\\ \approx \;\;1-\frac{S_{0}}{R\left(0\right)N}\left(1+\ln R\left(0\right)+\left(1-\frac{1}{\alpha }\right)\ln\frac{S\left(t_{1}\right)}{S_{0}}\right). \end{array}$$

Consequently, relative reduction in peak prevalence due to intervention within time *t*_1 _is *∈_α _*= (*I*(*t*_*m*,0_) *- I*(*t*_*m*,1_))/*N*, which can be parameterized as

(11) $${є}_{\alpha }=-\left(\frac{1}{\alpha }-1\right)\frac{S_{0}}{R\left(0\right)N}\ln\frac{S\left(t_{1}\right)}{S_{0}}.$$

Equation (11) indicates that the difference of peak prevalence between the two scenarios is determined by four different factors; the relative reduction in reproduction number *α *due to the intervention, initial condition at time 0, transmission potential *R*(0), and fraction of susceptible individuals at time *t*_1 _under the intervention. If the initial condition, the transmission dynamics in the absence of interventions (i.e. *R*(0), *β *and *γ*) and *t*_1 _are known, an estimate of *α *gives *S*(*t*_1_), yielding an estimate of ∈_*α*_.

### Delay in epidemic peak

The time to observe peak prevalence is analytically more challenging than the height of peak prevalence, because even an approximate estimate requires an analytical solution to the model (1). We propose a parsimonious approximation strategy which leads to more convenient solutions than those discussed in past studies (e.g. [29]). Substituting *I*(*t*) in the first sub-equation of (1) by (1/*γ*)(*dU*(*t*)/*dt*), we have

(12) $$\frac{1}{S\left(t\right)}\frac{dS\left(t\right)}{dt}=-\frac{\beta \left(t\right)}{\gamma }\frac{dU\left(t\right)}{dt}.$$

For the baseline scenario (i.e. *β*(*t*) = *β*), integrating (12) from time 0 to *t*,

(13) $$\ln\frac{S\left(t\right)}{S\left(0\right)}=-\frac{\beta }{\gamma }\left(U\left(t\right)-U\left(0\right)\right).$$

Because *U*(0)/*N *≈ 0,

(14) $$S\left(t\right)\approx S\left(0\right)\exp\left(-\frac{\beta }{\gamma }U\left(t\right)\right).$$

Subsequently, the third sub-equation of (1) is rewritten as

(15) $$\begin{array}{c} \frac{dU\left(t\right)}{dt}\;\;=\;\;\gamma \left(N-S\left(t\right)-U\left(t\right)\right),\\ \approx \;\;\gamma \left(N-S\left(0\right)\exp\left(-\frac{\beta }{\gamma }U\left(t\right)\right)-U\left(t\right)\right). \end{array}$$

Here we impose another key approximation. Because the quantity *βU*(*t*)/*γ *(*≈ R*(0)*U*(*t*)/*N*) for influenza (e.g. *R*(0) = 1.4) tends to be smaller than 1 (especially, before observing epidemic peak), we use a Taylor series expansion, i.e.,

(16) $$\exp\left(-\frac{R\left(0\right)U\left(t\right)}{S_{0}}\right)\approx 1-\frac{R\left(0\right)U\left(t\right)}{S_{0}}+\frac{1}{2}{\left(\frac{R\left(0\right)U\left(t\right)}{S_{0}}\right)}^{2}.$$

If the quadratic approximation is inadequate for large *R*(0), a higher order Taylor polynomial function can be used. Inserting the quadratic approximation into (15), and imposing a further approximation (i.e. *S*_0 _*≈ N*), we obtain

(17) $$\begin{array}{c} \frac{dU\left(t\right)}{dt}\;\;\approx \;\;\gamma \left(N-S\left(0\right)\left(1-\frac{R\left(0\right)U\left(t\right)}{S_{0}}+\frac{1}{2}{\left(\frac{R\left(0\right)U\left(t\right)}{S_{0}}\right)}^{2}\right)-U\left(t\right)\right),\\ \approx \;\;\gamma \left(R\left(0\right)-1\right)U\left(t\right)\left(1-\frac{R{\left(0\right)}^{2}}{2S_{0}\left(R\left(0\right)-1\right)}U\left(t\right)\right), \end{array}$$

which appears to be a logistic equation. We use this logistic-form solution instead of the more commonly employed hyperbolic-form solution [29,30], to illustrate a simpler approximate solution and to demonstrate the problem underlying both solutions. Later, we use a more formal solution (of logarithmic-form) in the intervention scenario, which is numerically identical to the classical hyperbolic-form solution (see below). Assuming that *U*(0) = *U*_0 _*>*0, the analytical solution of (17) is

(18) $$U\left(t\right)\approx \frac{U_{0}\exp\left(\gamma \left(R\left(0\right)-1\right)t\right)}{1+\frac{U_{0}R{\left(0\right)}^{2}}{2S_{0}\left(R\left(0\right)-1\right)}\left[\exp\left(\gamma \left(R\left(0\right)-1\right)t\right)-1\right]}.$$

The derivative of (18) is *dU*(*t*)/*dt *= *γI*(*t*). It follows that

(19) $$I\left(t\right)\approx \frac{U_{0}\left(R\left(0\right)-1\right)\left(1-\frac{U_{0}R{\left(0\right)}^{2}}{2S_{0}\left(R\left(0\right)-1\right)}\right)\exp\left(\gamma \left(R\left(0\right)-1\right)t\right)}{{\left[\frac{U_{0}R{\left(0\right)}^{2}}{2S_{0}\left(R\left(0\right)-1\right)}\left[\exp\left(\gamma \left(R\left(0\right)-1\right)t\right)-1\right]+1\right]}^{2}}$$

Further differentiation of (19) gives *dI*(*t*)/*dt*, and letting *dI*(*t*)/*dt *= 0, the time to observe peak prevalence is analytically derived. For the logistic equation, the corresponding time has been referred to as the inflection point of the cumulative curve in equation (18) [31]. The inflection point *t*_*m*,0 _to observe peak prevalence is

(20) $$t_{m,0}=\frac{1}{\gamma \left(R\left(0\right)-1\right)}\ln\left(\frac{2S_{0}\left(R\left(0\right)-1\right)}{U_{0}R{\left(0\right)}^{2}}-1\right),$$

which depends on initial condition and transmission characteristics. In the intervention scenario (in which intervention is short-lasting), an identical approach can be taken for *t < t*_1_, replacing *β *by *αβ *(or by replacing *R*(0) by *αR*(0)). Subsequently, the epidemic peak occurs at *t*_*m*,1 _(*> t*_1_). We take a similar approach to that used in (15) with a computed initial condition (*S*(*t*_1_), *I*(*t*_1_), *U*(*t*_1_)) using (18) and (19). For *t *≥ *t*_1_,

(21) $$\frac{dU\left(t\right)}{dt}=\gamma \left[N-S\left(t_{1}\right)\exp\left(-\frac{\beta }{\gamma }\left(U\left(t\right)-U\left(t_{1}\right)\right)\right)-U\left(t\right)\right],$$

Now we apply an approximation

(22) $$\exp\left(-\frac{\beta }{\gamma }\left(U\left(t\right)-U\left(t_{1}\right)\right)\right)\approx 1-\frac{R\left(0\right)}{S_{0}}\left(U\left(t\right)-U\left(t_{1}\right)\right)+\frac{1}{2}{\left(\frac{R\left(0\right)\left(U\left(t\right)-U\left(t_{1}\right)\right)}{S_{0}}\right)}^{2}.$$

It should be noted that, in the above approximation, we include the term exp(*βU*(*t*_1_)/*γ*), because *U*(*t*) *- U*(*t*_1_) better satisfies the Taylor series approximation than expanding *U*(*t*) alone. Let constants *A*, *B *and *C *be

(23) $$\begin{array}{l} A\;\;=\;\;\gamma \left(N-S\left(t_{1}\right)-\frac{R\left(0\right)S\left(t_{1}\right)U\left(t_{1}\right)}{S_{0}}-\frac{R{\left(0\right)}^{2}S\left(t_{1}\right)U{\left(t_{1}\right)}^{2}}{2S_{0}^{2}}\right),\\ B\;\;=\;\;\gamma \left(\frac{R\left(0\right)S\left(t_{1}\right)}{S_{0}}+\frac{R{\left(0\right)}^{2}S\left(t_{1}\right)U\left(t_{1}\right)}{S_{0}^{2}}-1\right),\\ C\;\;=\;\;\frac{\gamma S\left(t_{1}\right)}{2}{\left(\frac{R\left(0\right)}{S_{0}}\right)}^{2}. \end{array}$$

Given these constants, we consider

(24) $$\frac{dV\left(z\right)}{dz}=A+BV\left(z\right)-CV{\left(z\right)}^{2},$$

where *z *= *t - t*_1 _and *V *(*z*) = *U*(*z *+ *t*_1_) for *t *≥ *t*_1_. The initial condition *V *(0) is *V*_0 _= *U*(*t*_1_). Writing (24) in integral form, we have [30]

(25) $$\int_{V_{0}}^{V}\frac{1}{A+BV-CV^{2}}dV=\int_{0}^{z}dz.$$

Past studies have typically assumed hyperbolic-form functions for the analytical solution of (25) [29,30]. However, we express the solution in logarithmic-form [31,32], because logarithmic functions are compatible with spreadsheet programs. The logarithmic-form solution reads

(26) $$V\left(z\right)=\frac{\left(X+B\right)-Y\left(X-B\right)\exp\left(-Xz\right)}{2C\left(1+Y\exp\left(-Xz\right)\right)},$$

where

(27) $$X=\sqrt{B^{2}+4AC}.$$

Also,

(28) $$Y=\frac{X+B-2CV_{0}}{X-B+2CV_{0}}.$$

Differentiating (26) with respect to *z *and taking *dV *(*z*)/*dz *= 0, we find the inflection point *z*_*m*,1 _to be

(29) $$z_{m,1}=\frac{1}{X}\ln Y.$$

Replacing *z *by *t*, we obtain

(30) $$t_{m,1}=t_{1}+\frac{1}{\sqrt{B^{2}+4AC}}\ln\frac{X+B-2CU\left(t_{1}\right)}{X-B+2CU\left(t_{1}\right)},$$

as an approximate solution of the epidemic peak in intervention scenario. The time delay of this peak, imposed by the intervention in the early epidemic phase, *τ_α _*is subsequently calculated as

(31) $$\tau_{\alpha }=t_{m,1}-t_{m,0},$$

using (20) and (30) for the right-hand side. The delay depends on initial condition *U*_0_, the length of intervention *t*_1 _(both of which are apparent from (20) and (30)) and on the efficacy of intervention *α *(since this quantity influences the initial condition *U*(*t*_1_) in (30)).

### Application and illustration

#### Empirical analysis of influenza A (H1N1-2009)

**Figure 2:**
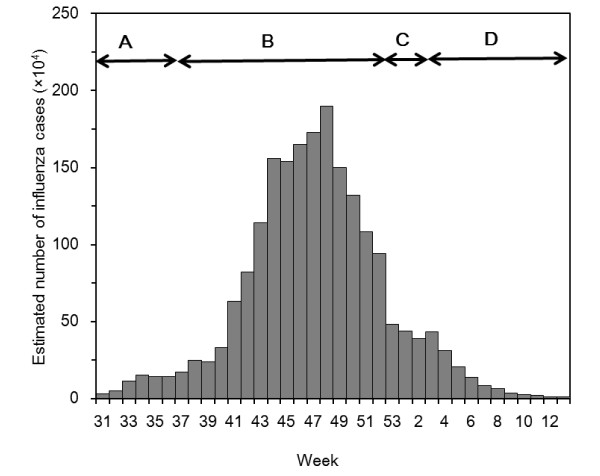
**Estimated weekly incidence of influenza cases in Japan from 2009-10**. The estimates are based on nationwide sentinel surveillance, covering the period from week 31 in 2009 to week 13 in 2010. The estimate follows an extrapolation of the notified number of cases from a total of 4800 randomly sampled sentinel hospitals to the total number of medical facilities in Japan. The case refers to influenza-like illness cases with medical attendance, possibly involving other diseases, but with influenza A (H1N1-2009) dominant among the isolated influenza viruses during the period of interest. Period A corresponds to summer school holiday, followed by autumn semester (period B). Period C covers winter holiday and period D corresponds to winter semester.

Here, we apply the above described theory to empirical influenza A (H1N1-2009) data. Figure 2 shows the estimated number of influenza cases based on national sentinel surveillance in Japan from week 31 (week ending 2 August) 2009 to week 13 (week ending 28 March) 2010. The estimates follow an extrapolation of the notified number of cases from a total of 4800 randomly sampled sentinel hospitals to the actual total number of medical facilities in Japan. The cases represent patients who sought medical attendance and who have met the following criteria, (a) acute course of illness (sudden onset), (b) fever greater than 38.0°C, (c) cough, sputum or breathlessness (symptoms of upper respiratory tract infection) and (d) general fatigue, or who were strongly suspected of the disease undertaking laboratory diagnosis (e.g. rapid diagnostic testing). Although the estimates of sentinel surveillance data involve various epidemiological biases and errors, we ignore these issues in the present study. Prior to week 31, the number of cases was small and the dynamics in the early stochastic phase have been examined elsewhere [17]. We arbitrarily assume that the major epidemic starts in week 31.

It is interesting to observe that the period A in Figure 2 corresponds to that of summer school holiday. Due to reporting delay of approximately 1 week [17], we assume that weeks 31 to 36 inclusive (the latter of which ends on 6 September) reflect the transmission dynamics during the summer school holiday. Subsequently, school opens in September with an epidemic peak in late November (period B), followed by abrupt decline during the winter holiday (period C) and start of winter semester (period D). Among these periods, we focus on the impact of summer holiday (period A), relative to period B, in lowering epidemic peak and delaying the time to observe the peak. More specifically, we estimate the reproduction number *R*(0) and its reduction *α *from the data set encompassing weeks 31 to 42. To permit an explicit estimation, we assume that linear approximation holds, as was similarly assumed elsewhere [15]. We assume that the reproduction number is reduced by a factor *α *from week 31 to 36 due to summer holiday, while the reproduction number recovers to *R*(0) from week 37 to 42.

Let *r*_0 _be the exponential growth rate of cases per day in the absence of summer holiday. Because our SIR model approximates the generation time by an exponential distribution with mean 1/*γ *days, the estimator of *R*(0) is [33,34]

(32) $$R\hat{(}0)=1+{\hat{r}}_{0}/\gamma .$$

Throughout the summer holiday, we assume that the reproduction number is reduced to *R_A _*= *αR*(0). That is, the growth rate during the summer holiday, *r*_1_, is defined by

(33) $$r_{1}=\alpha r_{0}+\gamma \left(\alpha -1\right).$$

Let the weekly incidence be *J_k _*in week *k*. During summer holiday period, the conditionally expected value of *J*_*k*+1 _given *J_k _*is

(34) $$\begin{array}{c} \text{E}\left(J_{k+1};J_{k}\right)\;\;=\;\;\exp\left(r_{1}\Delta t\right)J_{k},\\ =\;\;\exp\left[\left(\alpha r_{0}+\gamma \left(\alpha -1\right)\right)\Delta t\right]J_{k}, \end{array}$$

where Δ*t *is the length of reporting (i.e. 7 days). In week 37, the conditional expectation is

(35) $$\begin{array}{c} \text{E}\left(J_{k+1};J_{k}\right)\;\;=\;\;\frac{r_{1}\exp\left(r_{1}\Delta t\right)}{r_{0}}\frac{\exp\left(r_{0}\Delta t\right)-1}{\exp\left(r_{1}\Delta t\right)-1}J_{k},\\ =\;\;\frac{(\alpha r_{0}+\gamma \left(\alpha -1\right))\exp\left[\left(\alpha r_{0}+\gamma \left(\alpha -1\right)\right)\Delta t\right]}{r_{0}}\frac{\exp\left(r_{0}\Delta t\right)-1}{\exp\left[\left(\alpha r_{0}+\gamma \left(\alpha -1\right)\right)\Delta t\right]-1}J_{k}, \end{array}$$

because, with an initial incidence *i_k _*in week *k*, we have

(36) $$\text{E}\left(J_{k}\right)=i_{k}\int_{0}^{\Delta t}\exp\left(r_{1}t\right)dt=\frac{i_{k}}{r_{1}}\left(\exp\left(r_{1}\Delta t\right)-1\right),$$

and

(37) $$\text{E}\left(J_{k+1}\right)=i_{k}\exp\left(r_{1}\Delta t\right)\int_{0}^{\Delta t}\exp\left(r_{0}t\right)dt=\frac{i_{k}\exp\left(r_{1}\Delta t\right)}{r_{0}}\left(\exp\left(r_{0}\Delta t\right)-1\right).$$

See [35] for more details regarding the derivation of (35), which has been applied to influenza A (H1N1-2009) in other settings [36]. From week 38 to 42, E(*J*_*k*+1_; *J_k_*) simplifies to

(38) $$\text{E}(J_{k+\text{1}};J_{k})=\text{exp}(r_{0}\Delta t)J_{k}.$$

Although adding the information of test negative individuals could potentially yield a less biased estimate of *r*_0 _[37], we do not have access to this data and so we disregard this issue for now. Assuming that the observed counts of cases are Poisson distributed within each period, the likelihood function to estimate *r*_0 _and *α *is

(39) $$L\left(r_{0},\alpha ;J\right)=\prod_{k=32}^{42}\frac{\text{E}{\left(J_{k};J_{k-1}\right)}^{J_{k}}\exp\left(-\text{E}\left(J_{k};J_{k-1}\right)\right)}{J_{k}\;!}$$

The maximum likelihood estimates of *r*_0 _and *α *are obtained by minimizing the negative logarithm of (39), and the 95% confidence intervals (CI) are computed by profile likelihood. From the maximum likelihood estimates, we compute the differences in peak prevalence and times to observe the peak between baseline and second scenarios using the SIR model (1). For simplicity, we adopt (*S*_0_, *I*_0_, *U*_0_) = (99998, 1, 1) for the numerical computation and *t*_1 _is assumed to be 50 days (roughly corresponding to the length of period A plus 8 days).

Although our model (1) adopts exponentially distributed generation time, we can partially address the uncertainty of *r*_0 _and *α *in the parametric assumption of the generation time. That is, we adopt constant generation time (i.e. delta function) as an alternative assumption, which is known to yield a theoretical maximum reproduction number, given identical *r*_0 _and mean generation time [34]. Given *r*_0_, the estimator of *R*(0) with constant generation time of 1/*γ *days reads

(40) $$R\hat{(}0)=\exp\left(\frac{{\hat{r}}_{0}}{\gamma }\right).$$

As mentioned above, the reproduction number during summer holiday reduces to *R_A _*= *αR*(0). Accordingly, the growth rate during the summer holiday, *r*_1 _is written as

(41) $$r_{1}=r_{0}+\gamma \ln\alpha .$$

Using the above mentioned likelihood (39) and replacing *r*_1 _of exponential assumption by (41), we estimate *r*_0 _and *α*. It should be noted that the difference between (33) and (41) indicates that the estimates of both *r*_0 _and *α *depend on the realistic distribution of the generation time [25].

#### Sensitivity analysis

In addition to the analysis of empirical data, we also examine sensitivity of the height and time of peak prevalence to different values of *α *and *t*_1 _by numerical simulation. As mentioned above, influenza is our case study, and the default value of *R*(0) of baseline scenario is 1.4, but we also consider *R*(0) of 1.2 and 1.6. These ranges are adopted, additionally, because we impose an approximation (16). When obtaining the numerical solutions, initial condition is fixed at (*S*_0_, *I*_0_, *U*_0_) = (99998, 1, 1) for clarity. *U*_0 _= 1 is adopted to prevent *U*_0 _= 0 in (18) and in later equations, and also to select a positive integer value closest to 0 such that *U*_0_/*N ≈ *0.

## Results

### Influenza A (H1N1-2009)

**Figure 3:**
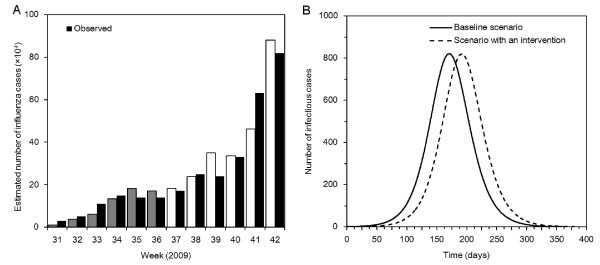
**The impact of summer holiday on the transmission dynamics of influenza A (H1N1-2009)**. **A**. Comparison of the observed and predicted weekly counts of the estimated number of influenza cases in Japan from week 31 to 42. Grey bars are conditionally expected values during the summer school holiday, while white bars are the conditionally expected values during autumn semester. **B**. Numerical solutions of the number of infectious individuals in a hypothetical population of 100,000 individuals with initial condition (*S*_0_, *I*_0_, *U*_0_) = (99998, 1, 1). Baseline scenario is compared against intervention scenario under a short-lasting intervention. For both scenarios, assumed *R*(0) and mean generation time are 1.14 and 3 days, respectively. In the second scenario, the transmission rate is reduced by a factor *α *= 0.948 due to summer school holiday from time 0 to *t*_1 _(=50 days). Although heights of peak prevalence do not greatly differ from each other, the time to observe the peak is clearly delayed in the second scenario.

Figure 3A compares observed and predicted numbers of influenza cases in Japan from week 31 to 42. Grey bars represent conditionally expected values during summer holiday, and white bars represent the expected values during autumn semester. The estimated growth rate in the absence of summer holiday, *r*_0 _is 0.048 (95% CI: 0.029, 0.066) per day. Thus, the estimated reproduction number *R*(0) is 1.14 (95% CI: 1.09, 1.20) which is likely an underestimate (see below).

The estimated relative proportion of the reproduction number under mitigation conditions *α *is 0.948 (95% CI: 0.842, 1.053). Although the confidence limits of *α *include 1, our argument adopts linear dynamics for longer than 10 weeks (note that the largest number of notifications is seen in week 48), and thus, the reproduction number is conservatively estimated (i.e. over the time period that we examine, the transmission dynamics may become nonlinear); therefore, *R*(0) is likely underestimated due to the linear approximation adopted in our quantitative illustration). Thus, we believe it is appropriate to regard the reduction in the reproduction number during summer holiday as marginally significant. The estimated reproduction number during school holiday is 1.08.

Even adopting constant generation time of 3 days, *r*_0 _is of a similar order, i.e. 0.048 (95% CI: 0.029, 0.066) per day. The reproduction number *R*(0), however, is slightly greater (1.15 (95% CI: 1.09, 1.22)) due to its estimator, exp(*r*_0_/*γ*). *α *is estimated at 0.942 (95% CI: 0.832, 1.061).

Figure 3B illustrates the number of infectious individuals in a hypothetical population with 100,000 individuals using the estimated *α *and *R*(0). In the absence of summer holiday, the epidemic peak would have been observed at Day 171 with *I*(*t*_*m*,0_) = 822 cases. In the presence of summer holiday from time 0 to *t*_1_, the peak is delayed to Day 192 with *I*(*t*_*m*,1_) = 820 cases. Thus, the estimated *α *and *R*(0) do not significantly alter the height of peak prevalence when the effects of summer holiday are included (a difference between the two scenarios of only 2 cases), but the delay effect between the scenarios is as long as 21 days. We do not use our approximate formula for the estimation of time delay in this empirical case study (for reasons explained below). Because *R*(0) = 1.14 in the absence of intervention, a major epidemic can occur when the initial condition *U*_0_/*N *is smaller than 1 - 1/*α R*(0) = 7.4% of the population. Assuming a fixed *I*_0 _= 1, and varying *S*_0 _and *U*_0 _from *N *- 1 to 0.926*N *and from 0 to 0.074*N*, respectively, only slight variations in the reduction of peak prevalence (not greater than 1 case) are observed, but the time delay varies greatly; under a scenario with *αR*(0) *≈ *1 or with *S*_0 _= 0.926*N *and *U*_0 _= 0.074*N*, a possible maximum delay of *t*_1 _= 50 days can be readily envisaged.

### Differential peak prevalence

**Figure 4:**
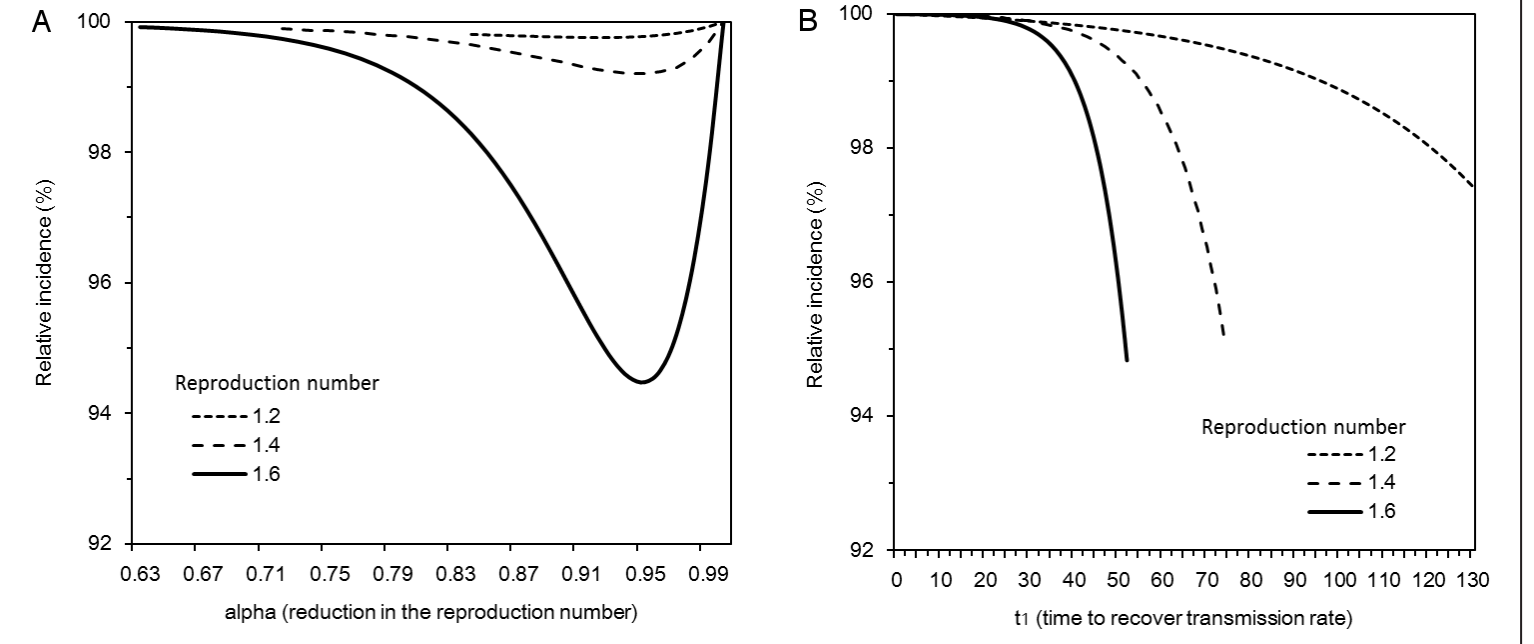
**Sensitivity analysis of peak prevalence to the efficacy and length of intervention**. **A**. Sensitivity of relative peak prevalence of infectious individuals to reduction in the reproduction number, *α*. The vertical axis represents *I*(*t*_*m*,1_)/*I*(*t*_*m*,0_), where *I*(*t*_*m*,0_) and *I*(*t*_*m*,1_) represent the peak prevalence in the absence and presence of intervention, respectively. The time length of intervention, *t*_1 _is fixed at Day 50. **B**. Sensitivity of relative peak prevalence of infectious individuals to the time length of the intervention, *t*_1_. *α *is fixed at 0.90. For both panels, the lines are truncated to satisfy *αR*(0) ≥ 1 and *t*_1 _<*t*_*m*,0 _where *t*_*m*,0 _is the time to observe peak prevalence in the absence of intervention.

Figure 4A examines the sensitivity of relative peak prevalence to *α *(i.e. reduction in *R*(0)) for assumed *R*(0) of 1.2, 1.4 and 1.6. Because *αR*(0) *<*1 prevents major epidemic during the early epidemic phase, possible ranges of *α *satisfying *αR*(0) ≥ 1 vary with *R*(0). It is worth noting that the relative reduction in peak prevalence is a nonlinear function of *α*. Largest reduction occurs when *α *lies within the range 0.90 to 0.95, rather than when *α *is minimum. Figure 4B examines the sensitivity of relative reduction in the peak prevalence as a function of the time length of intervention *t*_1 _(e.g. the time required to switch control policy from containment to mitigation). Again, to satisfy *t*_1 _<*t*_*m*,0_, possible ranges of *t*_1 _vary with *R*(0). The interpretation of Figure 4B is more straightforward than that of Figure 4A. Essentially, the longer the time period of intervention, the larger the potential reduction of prevalence.

The nonlinear relationship observed in Figure 4A can be explored by combining (11) with approximate solutions. First, because *S*(*t*_1_) *≈ S*(0) exp (-*α R*(0)*U*(*t*_1_)/*S*_0_) in the presence of intervention, equation (11) can be expressed in the form

(42) $$\in_{\alpha }=\left(1-\alpha \right)\frac{U\left(t_{1}\right)}{N}.$$

Second, using an approximate solution of *U*(*t*_1_) based on logistic equation (18),

(43) $$\in_{\alpha }\approx \frac{\left(1-\alpha \right)U_{0}(\exp\left(\gamma \left(\alpha R\left(0\right)-1\right)t_{1}\right)}{N+\frac{U_{0}{\left(\alpha R\left(0\right)\right)}^{2}N}{2S_{0}\left(\alpha R\left(0\right)-1\right)}\left[\exp\left(\gamma \left(\alpha R\left(0\right)-1\right)t_{1}\right)-1\right]},$$

which is a nonlinear function of *α*. Although the calculation is not shown here due to its mathematical complexity and lack of practical interpretation, the derivative of (43) with respect to *α *reveals an optimal *α *yielding the longest delay in Figure 4A.

### Delay in epidemic peak

**Figure 5:**
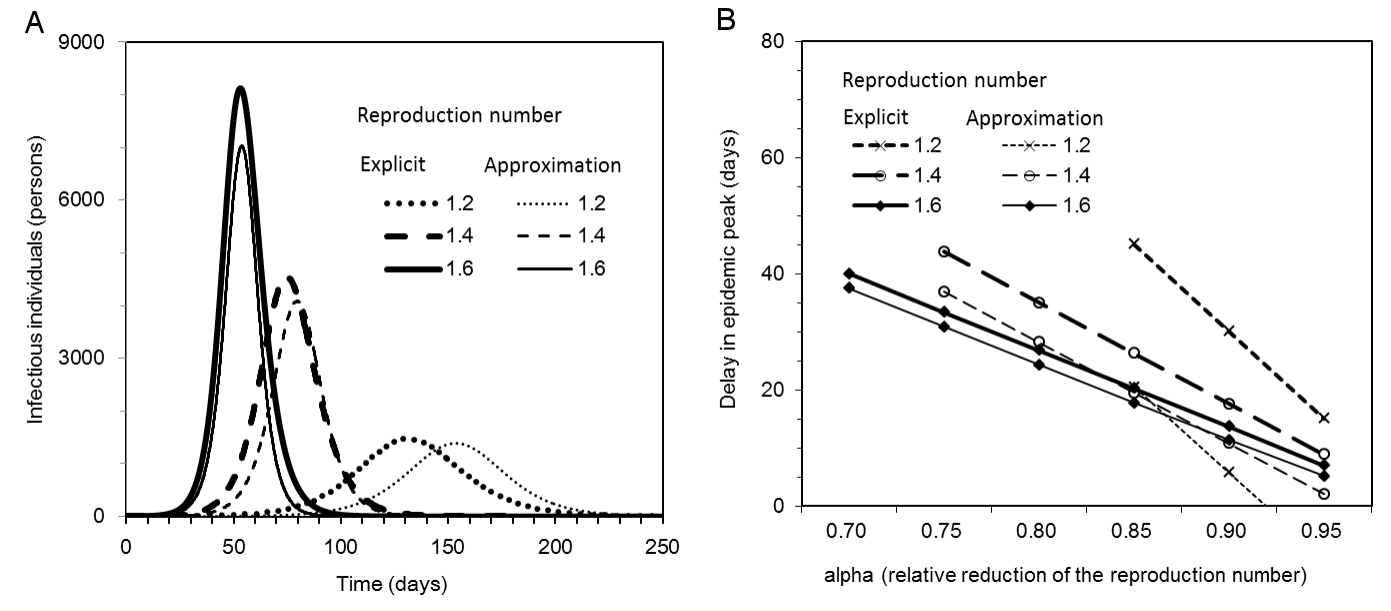
**Assessing approximation of time to observe epidemic peak**. **A**. Comparison of epidemic curves in the absence of intervention between explicit numerical and approximate solutions. The population size is assumed to be 100,000 with initial condition (*S*_0_, *I*_0_, *U*_0_) = (99998, 1, 1). The generation time is exponentially distributed with mean 1*/γ *= 3 days. **B**. Comparison of estimated delay in epidemic peak (imposed by a short-lasting intervention) between explicit numerical and approximate solutions. The assumed length of intervention *t*_1 _is 50 days.

Figure 5A compares epidemic curves of infectious individuals in the absence of intervention between explicit numerical and approximate solutions (i.e. solutions to (1) and (19), respectively). The height of epidemic peak is approximated well for smaller *R*(0), reflecting the fact that Taylor series expansion is a good approximation to the exponential function. The relationship between *R*(0) and approximation of epidemic peak height is also analytically expressed. Inserting (20) into (19), the approximate peak prevalence is

(44) $$I_{approx}\left(t_{m,0}\right)=\frac{S_{0}{\left(R\left(0\right)-1\right)}^{2}}{2R\left(0\right)}.$$

It should be noted that *S*_0_/*R*(0) can be replaced by *γ*/*β*, and thus, the peak prevalence in the approximated logistic form is independent of the initial condition (indeed, the derivative of a logistic equation is known not to depend on initial condition but rather on the carrying capacity [31]). On the other hand, an explicit solution can depend on initial condition for *U*(0) *>*0, i.e.,

(45) $$I\left(t_{m,0}\right)=\left(N-U_{0}\right)-\frac{\gamma }{\beta }\left(1+\ln R\left(0\right)\right).$$

These two quantities are identical for *R*(0) = 1, *N ≈ S*_0 _and *U*_0_/*N ≈ *0. Otherwise, numerical observation shows that *I*(*t*_*m*,0_) *> I_approx_*(*t*_*m*,0_) for *R*(0) *>*1 and *U*_0_/*N ≈ *0. Thus, the smaller the *R*(0), the better the approximate height of epidemic curve.

While interpretation of the height of peak prevalence is overall straightforward, the time to observe peak prevalence is better captured for larger *R*(0) (Figure 5A). Clearly, the logistic equation applied to *R*(0) = 1.2 yields a considerably biased (delayed) time to observation of epidemic peak (with bias longer than 20 days), and thus, we did not apply our approximate solution to the above mentioned case study of influenza A (H1N1-2009). It must be noted that the relationship between *R*(0) and approximation of epidemic peak in Figure 5A is regulated not only by *R*(0) but also by the initial condition *U*_0 _in (20); that is, the good agreement of the time of peak between two solutions for *R*(0) = 1.6 is not only due to *R*(0) but also to *U*_0 _= 1 in our simulation setting. For suitable values of *U*_0_, good approximations to the time of peak are obtained even for *R*(0) = 1.2 or smaller (results not shown).

Figure 5B compares the estimated time delay in epidemic peak, induced by an early intervention, between explicit numerical and approximate solutions. For all three *R*(0) that we investigate, approximation methods result in underestimation of the delay (31). For *R*(0) = 1.6, the approximate estimate of delay is crudely realized, and its sensitivity to *α *is close to that of the explicit numerical solution. The approximation is worst for *R*(0) = 1.2, for which a negative result was yielded for large *α*. These findings are in concordance with Figure 5A. It should be remembered that approximation of time of epidemic peak can vary with initial condition *U*_0_, indicating that the estimation requires knowledge of *U*_0 _in addition to *R*(0) and *α *(also, as we have seen, good approximation depends on judicious choice of *R*(0) and *U*_0_).

## Discussion

In the present study, we have presented fundamental ideas to assess the impact of a short-lasting intervention of an infectious disease on the epidemic peak. As a first step towards explicit evaluation of control effort in lowering and delaying the epidemic peak, we comprehensively described analytical expressions for the difference in the height of, and the time delay in, the epidemic peak gained by intervention, employing a parsimonious homogenous mixing epidemic model. We restricted our focus to a simple step function (Figure 1A) which adequately illustrated the role of summer holiday in lowering and delaying epidemic peak during the influenza (H1N1-2009) pandemic. Our methods show that both the height and the time of epidemic peak can be readily controlled by varying initial conditions at a given point of time at which transmission rate abruptly changes. The proposed method can be extended in future to encompass more realistic multiple steps for the transmission rate. Analytical solution of the simplest form of Kermack and McKendrick model has been undertaken multiple times, and is documented in many key references [27,29-32,38,39] including the original study in 1927 [13]. However, to our knowledge, the present study is the first to offer a theoretical basis on which to assess the impact of an early countermeasure on the epidemic peak employing logistic and logarithmic-form solutions, with a goal to making statistical inferences in the future. In addition, our analytical expressions not only promote epidemiological understanding of epidemic peak traits, but can be worked backwards to determine required control effort to achieve desired goals of height and delay of the epidemic peak. Although manual adjustment of the efficacy of intervention is not practically feasible, our demonstration of a nonlinear relationship between *α *and decline in the height of epidemic peak should be considered a key issue for optimal intervention management during an early epidemic phase.

Although public health guidelines have tended to advocate control policy switches from containment to mitigation at some point in time, the likely impact of unsuccessful containment to epidemic dynamics has been seldom discussed. Indeed, common illustration of mitigation (embodied in the three distinct goals (a)-(c) described in the Background section) do not account for the time-dependent transmission rate (rather, a guideline illustrates only differential peaks with various reproduction numbers *R*(0) [7]). Statistical modeling studies tend to focus only on time-dependent decreases in transmission potential, with a focus on the instantaneous reproduction number *R*(*t*) *<*1 to demonstrate successful control of an infectious disease (e.g. [40-42]). Motivated by these problems, we considered the impact of upward change in the transmission rate during the course of an epidemic. In our case study of influenza H1N1-2009, it was shown that summer vacation did not appreciably lower the height of an epidemic peak, but imposed a substantial time delay. Although our approximate estimation of the delay in epidemic peak was biased towards certain choices of *R*(0) and the initial number of immune individuals *U*_0_, this does not imply failure of the model. Rather, by means of approximate analytical computation, we have shown in (20) and (30) that the time of epidemic peak critically depends on the fraction of initially immune individuals prior to an epidemic. Although a preceding study instead emphasized the dependence of the time of peak on initial number of infectious individuals [32], other studies with hyperbolic-form solutions emphasize dependency of the time of peak on *U*_0 _[27,31]. We believe that *U*_0 _is more easily quantified than the initial number of infectious individuals *I*_0 _in practical settings. The critical importance of the initial number of immune individuals *U*_0 _is especially highlighted in the influenza A (H1N1-2009) pandemic because of pre-existing immunity [43-46]. Our analytical undertakings indicate that statistical inference of the time delay gained by early control effort will greatly benefit from population-wide seroepidemiological survey. Depending on the quality of approximation for a given combination of *R*(0) and *U*_0_, one can then decide whether the estimation of delay should be based on analytical or numerical solution.

Despite our motivation to eventually offer a method to estimate the impact of an early intervention, it should be noted that the present study does not account for uncertainty (e.g. confidence interval). Our arguments are based solely on deterministic models, whereas an explicit derivation of the confidence interval requires use of a stochastic Markov jump process [47]. Moreover, potential model extensions are numerous. Relevant factors include, but are not limited to, mobility of host, spatial dynamics, class-age structure (e.g. infection-age dependency), chronological age-structure, social contact patterns, seasonality, strain specificity and immunological dynamics. All of these features would increase the realism, but would greatly complicate analytical inspection, of the model. To illustrate the way forward, we discuss the simplest example of a class-age structured model in the Appendix.

Despite many future tasks to be completed, and our realization that the epidemic peak is vulnerable to heterogeneous patterns of transmission, relevant statistical assessment (including the estimation of *R*(0), generation time, and incubation period) always starts with a homogeneous modeling assumption [11,21,23,33,34,48,49]. This is particularly true during the early epidemic phase of a pandemic. In this sense, we believe that the present study has successfully offered a methodological avenue to statistically assess empirical data and to assess required control effort to lower and delay epidemic peak.

## Conclusions

This study has presented a theoretical basis on which to assess the impact of short-lasting intervention on the epidemic peak of an infectious disease. Employing a homogeneously mixing epidemic model, we derived analytical expressions for the decline in the height of epidemic peak and for the time delay of the peak. Empirical influenza A (H1N1-2009) data were analyzed using a simplistic but practically accessible model, which estimated that the epidemic peak was delayed for 21 days by the summer holiday period in 2009. Approximate logarithmic form solution of the time of epidemic peak appeared to critically depend on initial condition of immune individuals, supporting a need to conduct population-wide serological survey. Despite obvious needs to address various types of heterogeneity, our framework offers a successful methodological avenue to assess relevant empirical data and to advocate required control effort to lower and delay epidemic peak.

## Competing interests

The authors declare that they have no competing interests.

## Authors' contributions

HN conceived of the study, developed methodological ideas and implemented mathematical and statistical analyses. RO revised analytical results. HN drafted the manuscript and RO and HN discussed and revised the manuscript. All authors read and approved the final manuscript.

## Appendix: A way forward

In realistic situations, there is a time delay for newly infected individuals to acquire infectiousness, known as the latent period. This delay is captured by employing the so-called SEIR (susceptible-exposed-infected-recovered) model. In addition to *S*(*t*), *I*(*t*) and *U*(*t*), we consider infected but non-infectious individuals *E*(*t*). Assuming that the mean latent period is 1/*δ *days, the model is written as

(46) $$\begin{array}{l} \frac{dS\left(t\right)}{dt}\;\;=\;\;-\beta S\left(t\right)I\left(t\right),\\ \frac{dE\left(t\right)}{dt}\;\;=\;\;\beta S\left(t\right)I\left(t\right)-\delta E\left(t\right),\\ \frac{dI\left(t\right)}{dt}\;\;=\;\;\delta E\left(t\right)-\gamma I\left(t\right),\\ \frac{dU\left(t\right)}{dt}\;\;=\;\;\gamma I\left(t\right). \end{array}$$

In considering the height of epidemic peak of this system, an equation similar to (6) is derived by employing a Lyapunov function, *W*(*S*, *E*, *I*) [50]. A different derivation is given elsewhere [51].

(47) $$W\left(S,E,I\right)=S^{\left(-\frac{\gamma }{\beta }\right)}\exp\left(S+E+I\right).$$

The Lyapunov function is known to yield a constant solution (for any *t*), thus, *dW*/*dt *= 0. This is confirmed by

(48) $$\frac{dW\left(S,E,I\right)}{dt}=\frac{\partial W}{\partial S}\frac{dS}{dt}+\frac{\partial W}{\partial E}\frac{dE}{dt}+\frac{\partial W}{\partial I}\frac{dI}{dt}=0.$$

At an epidemic peak at time *t_m _*(where we have *dE*/*dt *= *dI*/*dt *= 0), two obvious conditions apply,

(49) $$S\left(t_{m}\right)\;\;=\;\;\frac{\gamma }{\beta },$$

(50) $$E\left(t_{m}\right)\;\;=\;\;\frac{\gamma }{\delta }I\left(t_{m}\right).$$

Using Lyapunov function in (47), we have

(51) $$W\left(S\left(t_{m}\right),E\left(t_{m}\right),I\left(t_{m}\right)\right)=W\left(S\left(0\right),E\left(0\right),I\left(0\right)\right).$$

Writing both sides of (51) in terms of (47), and taking logarithm of both sides, we obtain

(52) $$-\frac{\gamma }{\beta }\ln S\left(t_{m}\right)+S\left(t_{m}\right)+E\left(t_{m}\right)+I\left(t_{m}\right)=-\frac{\gamma }{\beta }\ln S\left(0\right)+S\left(0\right)+E\left(0\right)+I\left(0\right).$$

Rearranging (52) leads us to

(53) $$\left(1+\frac{\gamma }{\delta }\right)I\left(t_{m}\right)=S\left(0\right)+E\left(0\right)+I\left(0\right)-\frac{\gamma }{\beta }\left(1+\ln R\left(0\right)\right),$$

which is very close to (6). By varying initial conditions at each time point when a change in transmission rate occurs, equation (53) permits us to measure the impact of public health intervention on the epidemic peak, under the SEIR assumption. Regarding the time of epidemic peak, a straightforward extension of (15) applies, although the analytical solution may be rather complex or may not exist. We have

(54) $$\frac{dU\left(t\right)}{dt}=\gamma \left(N-S\left(t\right)-E\left(t\right)-U\left(t\right)\right).$$

*S*(*t*) can be replaced as for (14). Expressing *E*(*t*) as a function of *U*(*t*) requires strong mathematical supports. The problem may be addressed in different ways, but we first consider an analytical solution of *dE*/*dt*, i.e.,

(55) $$E\left(t\right)=E\left(0\right)\exp\left(-\delta t\right)+\exp\left(-\delta t\right)\int_{0}^{t}\exp\left(\delta \sigma \right)\left(-\dot{S}(\sigma )\right)d\sigma ,$$

where $\dot{S}(\sigma )$ in the right-hand side is equivalent to *dS*/*dσ *for which the derivative of approximation (15) can be used. We have yet to derive a simple analytical solution of the time of epidemic peak from (54), but the above discussion demonstrates that it is at least possible to compute the height of epidemic peak with more *E *compartments (as was discussed in [52]), using our proposed Lyapunov approach. Nevertheless, it should be remembered that the presence of infection-age dependency in the infectiousness profile is likely to complicate the computation of epidemic peak [53]. The incorporation of other realistic features, however, is greatly aided by the Lyapunov approach. In further studies, we will address epidemic peak with seasonality and age-dependent heterogeneity and in a multi-strain system.
